# miR-133a targets YES1 to reduce cisplatin resistance in ovarian cancer by regulating cell autophagy

**DOI:** 10.1186/s12935-021-02412-x

**Published:** 2022-01-10

**Authors:** Yang Zhou, Chunyan Wang, Jinye Ding, Yingying Chen, Yaoqi Sun, Zhongping Cheng

**Affiliations:** 1 Department of Gynecology and Obstetrics, Tenth People’s Hospital, Tongji University School of Medicine, Shanghai, 200072 China; 2 Institute of Gynecological Minimally Invasive Surgery Research Center, Tongji University School of Medicine, Shanghai, 200072 China

**Keywords:** miR-133a, Ovarian cancer, YES1, Cisplatin resistance, Autophagy

## Abstract

**Background:**

Accumulating evidence has revealed that aberrant microRNA (miRNA) expression can affect the development of chemotherapy drug resistance by modulating the expression of relevant target proteins. Emerging evidence has demonstrated that miR-133a participates in the tumorigenesis of various cancers. However, whether miR-133a is associated with cisplatin resistance in ovarian cancer remains unclear.

**Objective:**

To investigate the role of miR-133a in the development of cisplatin resistance in ovarian cancer.

**Methods:**

MiR-133a expression in cisplatin-resistant ovarian cancer cell lines was assessed by reverse-transcription quantitative PCR (RT–qPCR). A cell counting kit-8 (CCK-8) assay was used to evaluate the viability of tumour cells treated with cisplatin in the presence or absence of miR-133a. A luciferase reporter assay was used to analyse the binding of miR-133a with the 3′ untranslated region (3′UTR) of YES proto-oncogene 1 (YES1). The YES1 expression level was analysed using a dataset from the International Cancer Genome Consortium (ICGC) and assessed by RT–qPCR and western blotting in vitro. The roles and mechanisms of YES1 in cell functions were further probed via gain- and loss-of-function analysis.

**Results:**

The expression of miR-133a was significantly decreased in cisplatin-resistant ovarian cancer cell lines (A2780-DDP and SKOV3-DDP), and the overexpression of the miR-133a mimic reduced cisplatin resistance in A2780-DDP and SKOV3-DDP cells. Treatment with the miR-133a inhibitor increased cisplatin sensitivity in normal A2780 and SKOV3 cells. MiR-133a binds the 3’UTR of YES1 and downregulates its expression. Bioinformatics analysis revealed that YES1 expression was upregulated in recurrent cisplatin-resistant ovarian cancer tissue, and in vitro experiments also verified its upregulation in cisplatin-resistant cell lines. Furthermore, we discovered that miR-133a downregulated the expression of YES1 and thus inhibited cell autophagy to reduce cisplatin resistance. Yes1 knockdown significantly suppressed the cisplatin resistance of ovarian cancer cells by inhibiting autophagy in vitro. Xenograft tumour implantation further demonstrated that Yes1 overexpression promoted ovarian tumour development and cisplatin resistance.

**Conclusions:**

Our results suggest that the miR-133a/YES1 axis plays a critical role in cisplatin resistance in human ovarian cancer by regulating cell autophagy, which might serve as a promising therapeutic target for ovarian cancer chemotherapy treatment in the future.

**Supplementary Information:**

The online version contains supplementary material available at 10.1186/s12935-021-02412-x.

## Background

Ovarian cancer is one of the most lethal gynaecologic cancers that seriously threaten women’s health [1]. In the past 30 years, comprehensive/systematic surgery followed by platinum-based chemotherapy has remained the main treatment, but the overall prognosis of ovarian cancer has not improved significantly, and the 5-year survival rate of patients with advanced (FIGO 3–4) ovarian cancer is less than 30% [2]. Tumour relapse and acquired drug resistance are treatment bottlenecks for improving the overall survival of ovarian cancer patients [3], and the molecular mechanism of drug resistance in ovarian cancer remains unknown. Thus, further understanding of the pathogenesis and mechanisms of ovarian cancer chemotherapy resistance is crucial to improve overall survival, identify therapeutic markers, and develop new efficient treatment strategies.

Aberrant microRNA (miRNA) expression plays critical roles in various types of cancers, and some of them are considered ideal targets for tumour treatment [4]. Emerging evidence has demonstrated that miR-133a participates in the tumorigenesis of various cancers [5]. For example, Wang reported that miR-133a expression was downregulated in pancreatic cancer tissues and serum specimens of patients, and its expression was negatively correlated with the stage and prognosis of pancreatic cancer patients, which may be an ideal marker for the early diagnosis of pancreatic cancer [6]. Yuan et al. reported that the nuclear paraspeckle assembly transcript 1 (NEAT1)/miR133a axis promoted cervical cancer progression by regulating SRY-box transcription factor 4 (SOX4) [7]. In addition, miR-133a was expressed at low levels in gastric cancer tissues and cells, and it inhibited tumour cell proliferation and metastasis by regulating the autophagy of gastric cancer tumour cells [8]. Our previous research found that the miR-133a/glycogen phosphorylase B (PYGB) axis can inhibit the occurrence and development of ovarian cancer both in vitro and in vivo [9]. Moreover, miR-133a also plays a key role in tumour drug resistance. Overexpression of miR-133a decreased oxorubicin resistance in the MCF-7/Dox breast cancer cell line [10]. MiR-133a could reduce cisplatin resistance in non-small-cell lung cancer cells and Hep-2v cells [11, 12]. However, the function of miR-133a in ovarian cancer chemotherapy resistance has not been researched. YES1 functions as a tumour oncogene and may be a potential therapeutic target in different types of cancers [13]. Knockdown of YES1 led to the suppression of proliferation and cell cycle arrest in ovarian cancer cells [14]. A clinical retrospective study showed that overexpression of Yes1 indicated favourable prognosis and increased platinum sensitivity in primary epithelial ovarian cancer patients [15]. Moreover, the expression of YES1 was higher in recurrent postchemotherapy high-grade serous ovarian cancer. Overexpression of Yes1 decreased the rate of apoptotic cells in OVCAR8 cells with Taxol treatment, suggesting a potential association between YES1 and Taxol chemoresistance [16]. However, how YES1 regulates chemoresistance in recurrent ovarian cancer is not fully understood.

In this study, we aimed to explore the expression pattern and functional role of miR-133a in cisplatin-resistant ovarian cancer and its potential mechanism in regulating cisplatin resistance in ovarian cancer. We found that miR-133a is downregulated in cisplatin-resistant ovarian cancer and that overexpression of miR-133a could reduce cisplatin resistance in ovarian cancer cell lines. Mechanistically, miR-133a downregulates YES1 by binding to the 3′UTR of YES1. Knockdown of Yes1 inhibits cisplatin resistance in a cisplatin-resistant cell line, and overexpression of Yes1 induces cisplatin resistance in cisplatin-sensitive cells. Furthermore, we demonstrated that YES1 affects cisplatin resistance by regulating autophagy in both ovarian cancer cells and a xenograft model. Taken together, our findings suggest that miR-133a targets the 3′UTR of YES1 and reversely regulates the expression of YES1, which ultimately regulates cell autophagy in ovarian cancer with cisplatin resistance.

## Materials and methods

### Cell culture and treatment

Human ovarian cancer cells (SKOV3 and A2780) were obtained from the Cell Bank, Shanghai Institute of Biochemistry and Cell Biology (Shanghai, China). Cisplatin-resistant cell lines (SKOV3-DDP and A2780-DDP) were established in our lab. All cell lines were cultured in RPMI 1640 medium (Gibco) supplemented with 10% (v/v) foetal bovine serum (FBS, Gibco), 100 µg/ml streptomycin, and 100 U/ml penicillin in a humidified atmosphere at 37 °C with 5% CO_2_. For cisplatin treatment, ovarian cancer cells were cultured with 10–80 µM cisplatin (selleck) for 48 h. For autophagic promotion and inhibition, cells were treated with the autophagic antagonist chloroquine (50 µM, Sigma) and the autophagic agonist rapamycin (100 nM, Sigma), respectively.

### qRT–PCR

Total RNA from tissues and cultured cells was extracted using TRIzol reagent (Sigma). RNA concentration was measured and equal amount of mRNA was reverse transcribed to cDNA using reverse transcription kits (Takara), meanwhile miRNA was reverse transcribed to cDNA using reverse transcription kits (TransGen). Quantitative real-time PCR (qPCR) was performed on an ABI 7500 real-time PCR system using the following specific primers: YES1 forwards primer: 5′-CTCAGGGGTAACGCCTTTTGG-3′; reverse primer: 5′-CACCACCTGTTAAACCAGCAG-3′; GAPDH forwards primer: 5′-GGAGCGAGATCCCTCCAAAAT-3′; reverse primer: 5′-GGCTGTTGTCATGCTTCTCATGG-3′; miR-133a forwards primer: 5′-CAGCTGGTTGAAGGGGACCAAA-3′; U6 forwards primer: 5′-CTCGCTTCGGCAGCACA-3′; reverse primer: 5′-AACGCTTCACGAATTTGCGT-3′. GAPDH was used as an internal control for mRNA, and U6 was used as an internal control for miRNA. Relative YES1 expression was normalized to GAPDH levels, and relative miR-133a was normalized to U6 levels using the 2^−ΔΔCt^ quantification method.

### YES1 and miR-133a transfection

YES1-overexpression (YES1-OE) vectors and a negative control and miR-133a mimics, miR-133a inhibitor and relative controls were purchased from Yazai Biotechnology Co Ltd. (Shanghai, China). SiYES1 and relative controls were purchased from Gene Pharma (Shanghai, China). Transfection was conducted using Lipofectamine 3000 according to the manufacturer’s manual. Lentivirus-YES1-OE for stable overexpression of YES1 or the negative control construct was designed and constructed by Hanbio Biotechnology Co., Ltd. (Shanghai, China).

### Western blot analysis

Tissue samples or cultured cells were lysed in RIPA buffer (CWBIO, CW2333S), and the protein concentration was measured by the BCA protein assay. Equal amounts of protein were resolved by 10% or 15% SDS–PAGE and transferred onto a polyvinylidene fluoride (PVDF) membrane. The membranes were blocked at room temperature with 5% skimmed milk and then incubated with primary antibodies overnight at 4 °C. Primary antibodies against YES1 (1:1000, ABclonal, A0628), LC3b (1:1000, Abcam, AB192890), and Gapdh (1:5000, Proteintech, 51067-2-AP) were used. After washing with TBST (Tris-buffered saline with Tween), the membranes were further incubated with fluorescent secondary antibodies (1:5000, ABclonal, AS014) at room temperature in the dark for 1-2 h. Then, the membranes were detected by an enhanced chemiluminescence detection kit (Epizyme, SQ202). GAPDH was used as internal control. The signals were detected using an Odyssey detection system (Odyssey CLx, LI-COR biosciences, NE, USA). Quantification analysis of western blots was performed using ImageJ software (Bethesda, USA).

### Cell proliferation

Cell proliferation was determined by Cell Counting Kit-8 assay (CCK-8) (Dojindo Laboratories, Kumamoto) according to the manufacturer’s protocol. SKOV3 and SKOV3-DDP cells were seeded at a density of 3 × 10^3^ cells/well, and A2780 and A2780-DDP cells were seeded at a density of 8 × 10^3^ cells/well into a 96-well plate. Ten microlitres of CCK-8 working solution was added, and the cells were incubated for 2 h before the absorbance was measured at 450 nm.

### Luciferase reporter assays

WT or mutated 3′-UTR of YES1 sequences were cloned and constructed into the pGL3-Luc reporter vector. A2780 cells were transfected with WT or mutated luciferase reporter vectors together with miR-133a mimics or negative control. Relative luciferase activity was analysed using a Dual-Luciferase Reporter Assay Kit 48 h later.

### GFP-RFP-LC3 assay

To monitor autophagy, the tandem GFP-RFP-LC3 adenovirus construct obtained from Hanbio Inc. (Shnaghai, China), which capitalizes on the pH difference between the acidic autolysosome and the neutral autophagosome and the pH sensitivity differences exhibited by GFP (green fluorescent protein) and RFP (red fluorescent protein) to monitor progression from the autophagosome to autolysosome, was used. In brief, A2780 and A2780-DDP cells were infected with tandem GFP-RFP-LC3 adenovirus for 2 h and then cultured with normal medium and 10 µM cisplatin for 48 h. Finally, cells were treated and imaged for GFP and RFP by using fluorescence microscopy.

### International Cancer Genome Consortium (ICGC) dataset analysis

The RNA sequencing data of ICGC OV-AU used for gene expression analysis. Genes with low read abundance were filtered, followed by rlog transformation based on the R package DESeq2 (version 1.28.1). Then, principal component analysis (PCA) of the normalized expression matrix, relied on the FactoMineR package (version 2.3), was performed to detect the outlying samples. The remaining samples were randomly divided into primary sensitive (n = 12) and relapse resistant (n = 24) groups from 24 high-grade serous ovarian cancer (HGSOC) patients [17].

### In vivo tumorigenicity

All mouse experiments were approved by the Experimental Animal Ethics Committee of the Tenth People’s Hospital Affiliated with Tongji University. Female BALB/c nude mice (4–5 weeks old, 18–20 g) were purchased from Vital River Laboratory (Beijing, China). For the xenograft model, 5 × 10^6^ A2780 ovarian cancer cells transfected with lentivirus-YES1-OE or the negative control were subcutaneously implanted into nude mice. Intraperitoneal injection with a density of 5 mg/kg cisplatin was conducted every 3 Days a week after all the tumour nodules in the same group appeared. Tumour volume was calculated based on tumour sizes determined by a Vernier caliper every week (length × width^2^/2). The mice were sacrificed by cervical dislocation 14 days later. Tumour samples were collected, and tumour weights were determined.

### Statistical analysis

All statistical analyses were conducted using SPSS software (version 23.0) and GraphPad Prism 6. Experimental results are presented as the mean ± standard deviation of the mean (SD) based on the results from three independent experiments. Unpaired two-tailed Student’s t test and one-way analysis of variance (ANOVA) were used where necessary for calculation of p values. Differences in clinicopathological factors between the YES1 high- or low-expression groups were analysed via the Chi-square test. P values < 0.05 were considered statistically significant.

## Results

### MiR-133a can reduce cisplatin resistance in ovarian cancer cell lines

**Fig. 1:**
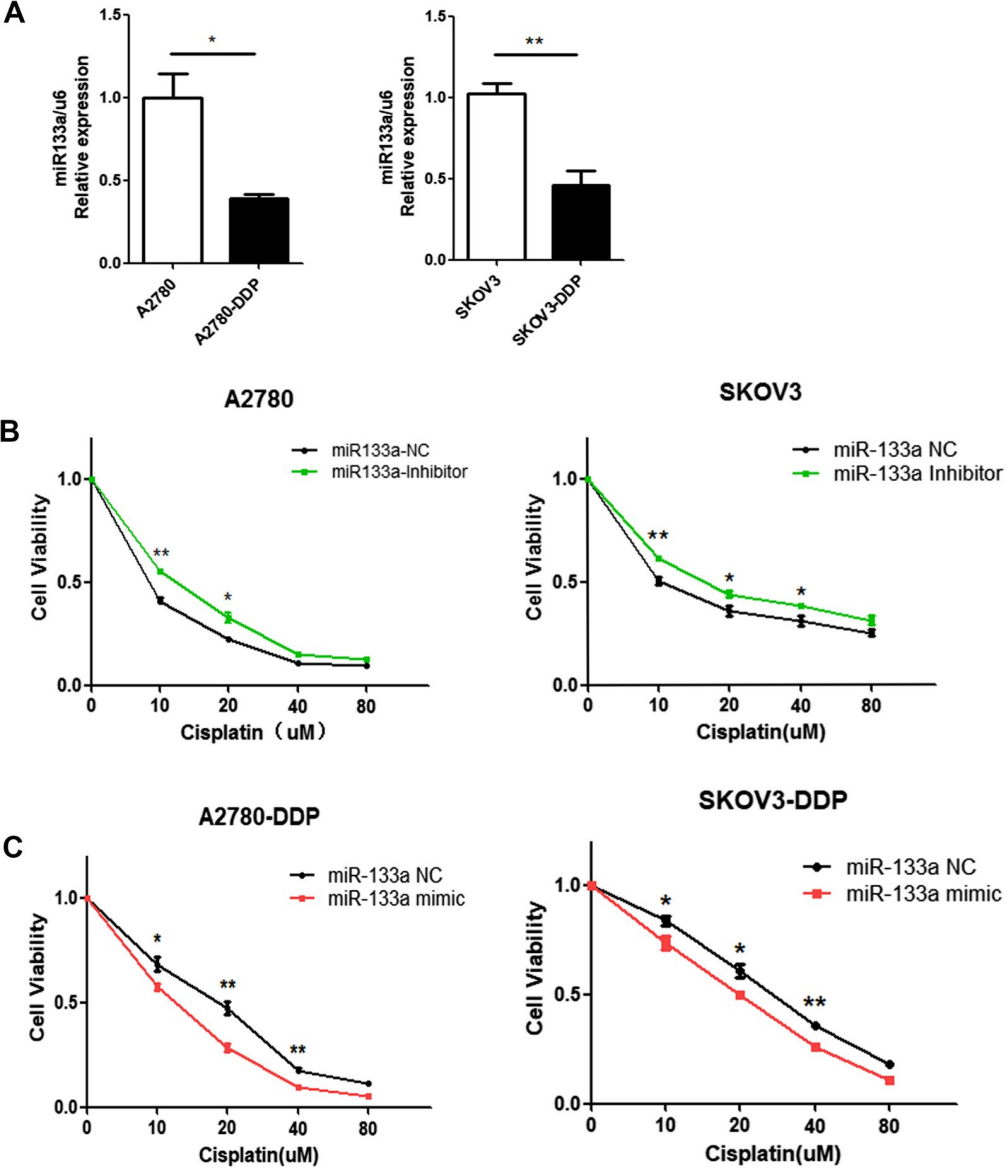
MiR-133a can reduce cisplatin resistance in cisplatin-resistant ovarian cancer cell lines. **A** MiR-133a is downregulated in SKOV3-DDP and A2780-DDP cell lines compared with SKOV3 and A2780 cells, respectively. **B** MiR-133a knockdown induced cisplatin resistance in A2780 and SKOV3 cells. **C** MiR-133a mimic increased cisplatin sensitivity in A2780-DDP and SKOV3-DDP cell lines. For comparisons, Student’s t test was performed; *p < 0.05, **p < 0.01, ***p < 0.001

To determine the expression of miR-133a in cisplatin-resistant ovarian cancer cells, we examined the expression level of miR-133a in A2780-DDP and SKOV3-DDP cells compared with SKOV3 and A2780 cells by RT–qPCR, and the results showed that the miR-133a expression level was remarkably lower in cisplatin-resistant ovarian cancer cell lines (A2780-DDP, SKOV3-DDP) than in parental cisplatin-sensitive ovarian cancer cells (A2780, SKOV3) (Fig. 1A). To further verify the function of miR-133a in cisplatin sensitivity, we disturbed the miR-133a function in ovarian cancer cell lines, and the results showed that treatment with the miR-133a inhibitor increased cisplatin resistance in A2780 and SKOV3 cells (Fig. 1B), and miR-133a mimic treatment increased cisplatin sensitivity in A2780-DDP and SKOV3-DDP cells (Fig. 1C). These findings suggested that miR-133a was downregulated in cisplatin-resistant ovarian cancer cells and may reduce cisplatin resistance.

### MiR-133a regulated YES-1 by binding to the 3′-UTR of YES1

**Fig. 2:**
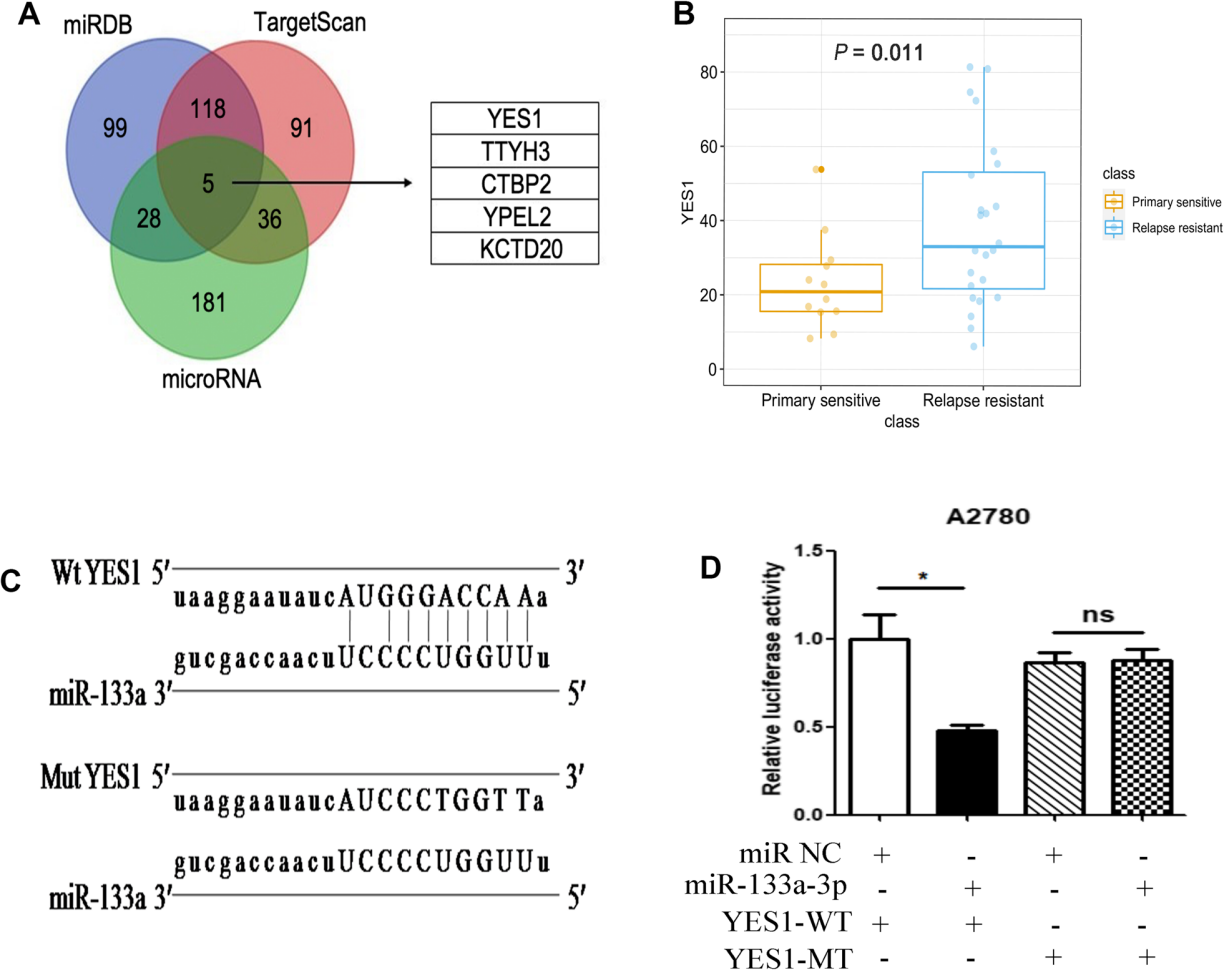
MiR-133a targets YES1 by binding to the 3′-UTR of YES1. **A** YES1, TTYH3, CTBP2, YPEL2, and KCTD20 were selected as potential downstream genes regulated by miR-133a by intersection analysis from 3 databases (miRDB, TargetScan, and microRNA). **B** Among the 5 potential genes, the YES1 expression level was dramatically increased in drug-resistant ovarian cancer tissues compared with primary ovarian cancer tissues. **C** The predicted miR-133a binding sequence in the 3’UTR of YES1 and the generation of dual-luciferase reporter plasmids of YES1-Wt and YES1-Mut are shown. **D** Luciferase activity assays were carried out in A2780 cells cotransfected with miR-133a mimic or miR-133a NC and YES1-Wt or YES1-Mut. For comparisons, Student’s t test was performed; *p < 0.05

To understand how miR-133a regulates cisplatin resistance in ovarian cancer, we performed bioinformatics analysis using a miRBase online tool to predict the potential genes regulated by miR-133a (Fig. 2A). Five potential genes had putative 3′-UTR binding sites that matched the miR-133a sequences by intersection analysis from 3 databases (miRDB, TargetScan, and microRNA). To further understand which target gene among the 5 potential genes affects the chemotherapy resistance of ovarian cancer cells, we analysed the gene expression level between the primary ovarian cancer and recurrent ovarian cancer groups, and the results demonstrated that only the YES1 expression level was dramatically increased in recurrent ovarian cancer tissues compared with that in primary tissues (Fig. 2B and Additional file 1: Fig. S1). We next investigated the association between miR-133a and YES1 through luciferase activity analysis in A2780 cells transfected with a luciferase vector containing the predicted miR-133a binding sites (Fig. 2C). Our data demonstrated that the luciferase activity of the reporter including the WT YES1 3′UTR sequence was significantly reduced by miR-133a mimic treatment, and mutation of this sequence abolished the negative effects (Fig. 2D).

### Yes1 knockdown reduced cisplatin resistance in vitro

**Fig. 3:**
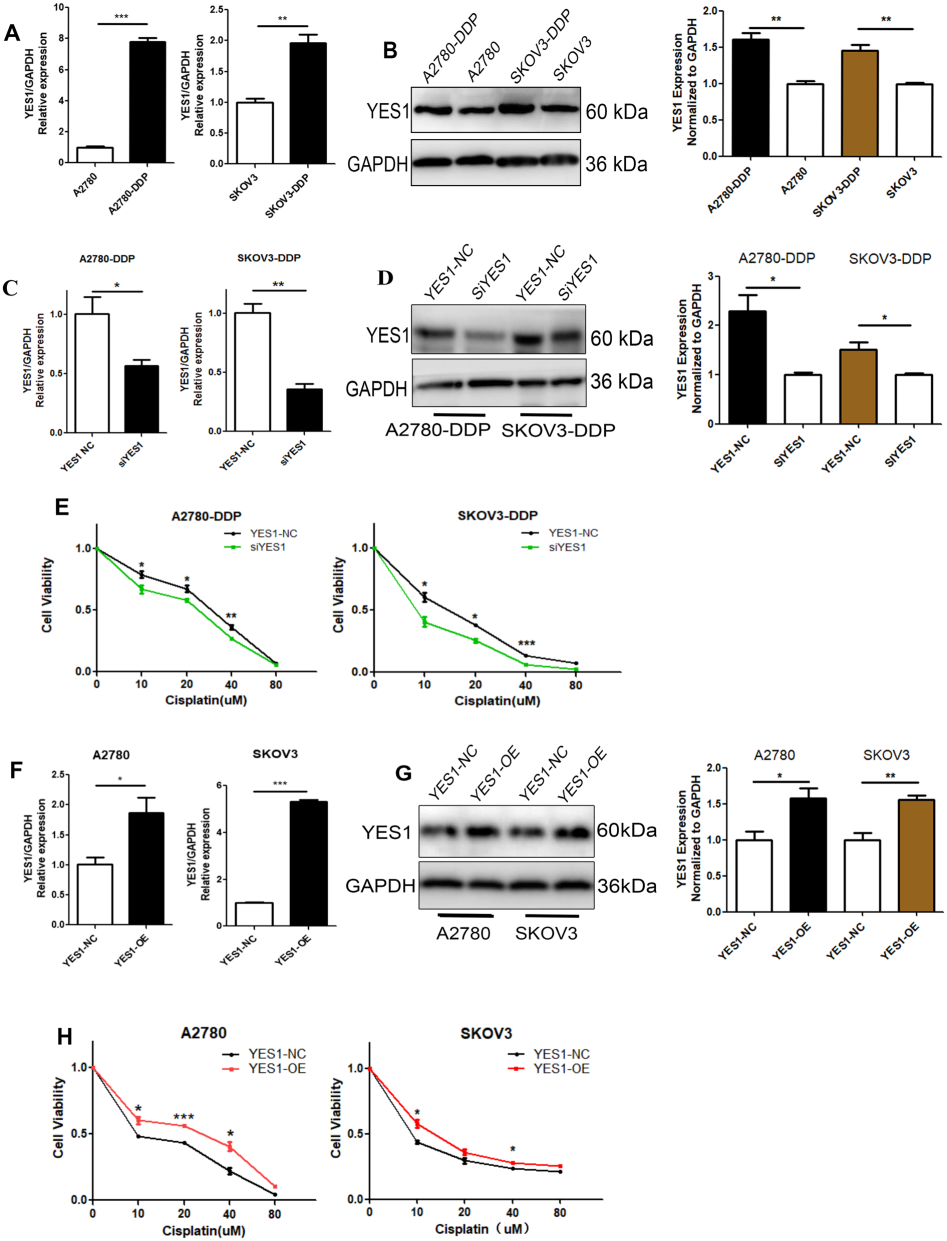
YES1 depletion decreases cisplatin sensitivity in ovarian cancer cell lines. **A** and **B** YES1 was significantly upregulated at both the mRNA level (**A**) and protein level (**B**) in A2780-DDP and SKOV3-DDP cell lines compared with A2780 and SKOV3 cells, respectively. **C** and **D** YES1 mRNA levels (**C**) and protein expression (**D**) were reduced significantly after siRNA targeting YES1 transfection. **E** Yes1 knockdown increases cisplatin sensitivity in A2780-DDP and SKOV3-DDP cell lines. **F** and **G** YES1 mRNA levels (**F**) and protein expression (**G**) were increased significantly after YES1-OE vector transfection. **H** Yes1 overexpression increases cisplatin resistance in A2780 and SKOV3 cells. For comparisons, Student’s t test was performed; *p < 0.05, **p < 0.01

To further understand how YES1 plays a role in cisplatin resistance in ovarian cancer, we first demonstrated that YES1 was significantly highly expressed in both A2780-DDP and SKOV3-DDP cells by RT–qPCR and WB (Fig. 3A and B), which is consistent with the results of bioinformatics analysis (Fig. 2B). To further explore the function of YES1 affecting cisplatin sensitivity in ovarian cancer, we screened for a highly efficient Yes1 knockdown siRNA by measuring the silencing efficiency of three YES1 siRNAs with different targeting sequences in SKOV3-DDP or A2780-DDP cells (Additional file 1: Fig. S2), si-YES1#2 (referred to as siYES1 in the subsequent experiments) transfection in A2780-DDP and SKOV3-DDP ovarian cancer cells significantly downregulated YES1 expression at both the mRNA and protein levels (Fig. 3C and D). We found that knockdown of Yes1 significantly reduced cisplatin resistance in SKOV3-DDP and A2780-DDP cells (Fig. 3E). Meanwhile, Yes1-OE enhanced cisplatin resistance in SKOV3 and A2780 cell lines (Fig. 3F–H).

### MiR-133a inhibit the expression of YES1

**Fig. 4:**
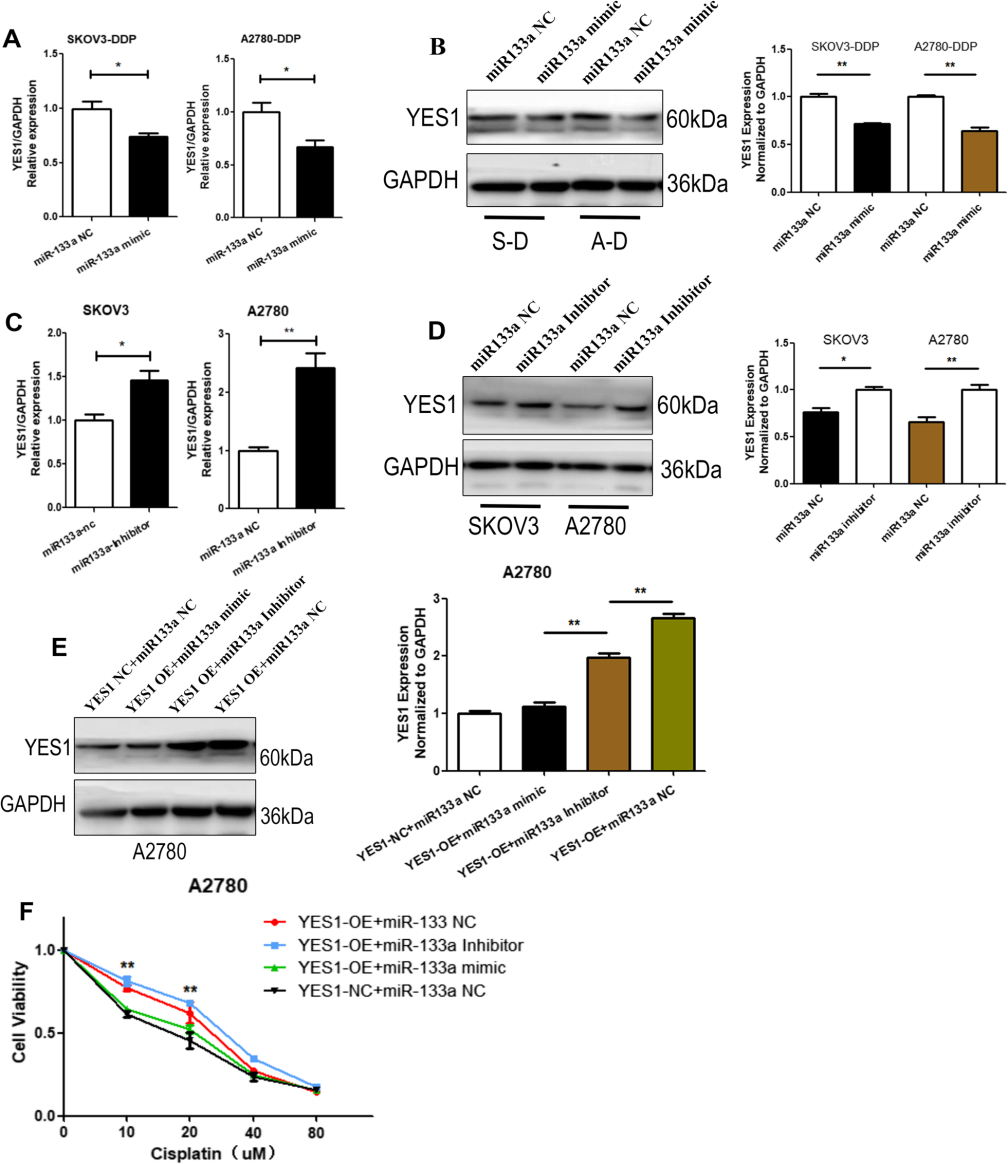
MiR-133a targets YES1 and downregulates its expression. **A** and **B** The miR-133a mimic inhibited YES1 expression in SKOV3-DDP and A2780-DDP cells at both the mRNA (**A**) and protein (**B**) levels. **C** and **D** MiR-133a inhibitor upregulated YES1 expression in SKOV3 and A2780 cells at both the mRNA (**C**) and protein (**D**) levels. **E** The miR-133a mimic reduced the YES1 overexpression function induced by YES1-OE, but the miR-133a inhibitor had no effect on the overexpression of YES1 compared with the miR-133a NC + YES1-OE group. **F** Cotransfection of the YES1-OE vector together with miR-133a mimic transfection antagonized YES1-OE-induced cisplatin resistance. For comparisons, Student’s t test was performed; *p < 0.05, **p < 0.01

To further evaluate the molecular mechanism, miR-133a was overexpressed by treatment with miR-133a mimics, which significantly inhibited YES1 expression in SKOV3-DDP or A2780-DDP cells at both the mRNA and protein levels (Fig. 4A and B). Similarly, miR-133a inhibitor treatment increased YES1 expression in SKOV3 and A2780 cells at both the mRNA and protein levels (Fig. 4C and D). Furthermore, cotransfection of the miR-133a mimic and YES1-OE vector decreased YES1 expression compared with that in the miR-133a NC + YES-OE group, but the miR-133a inhibitor did not affect YES1 expression (Fig. 4E). Functionally, we demonstrated that overexpression of YES1 together with miR-133a mimic transfection antagonized YES-OE-induced cisplatin resistance (Fig. 4F). Taken together, these findings suggested that miR-133a targets YES1 and downregulates its expression, thereby reducing cisplatin resistance in ovarian cancer cells.

### YES1 regulates autophagy in cisplatin-resistant ovarian cancer

**Fig. 5:**
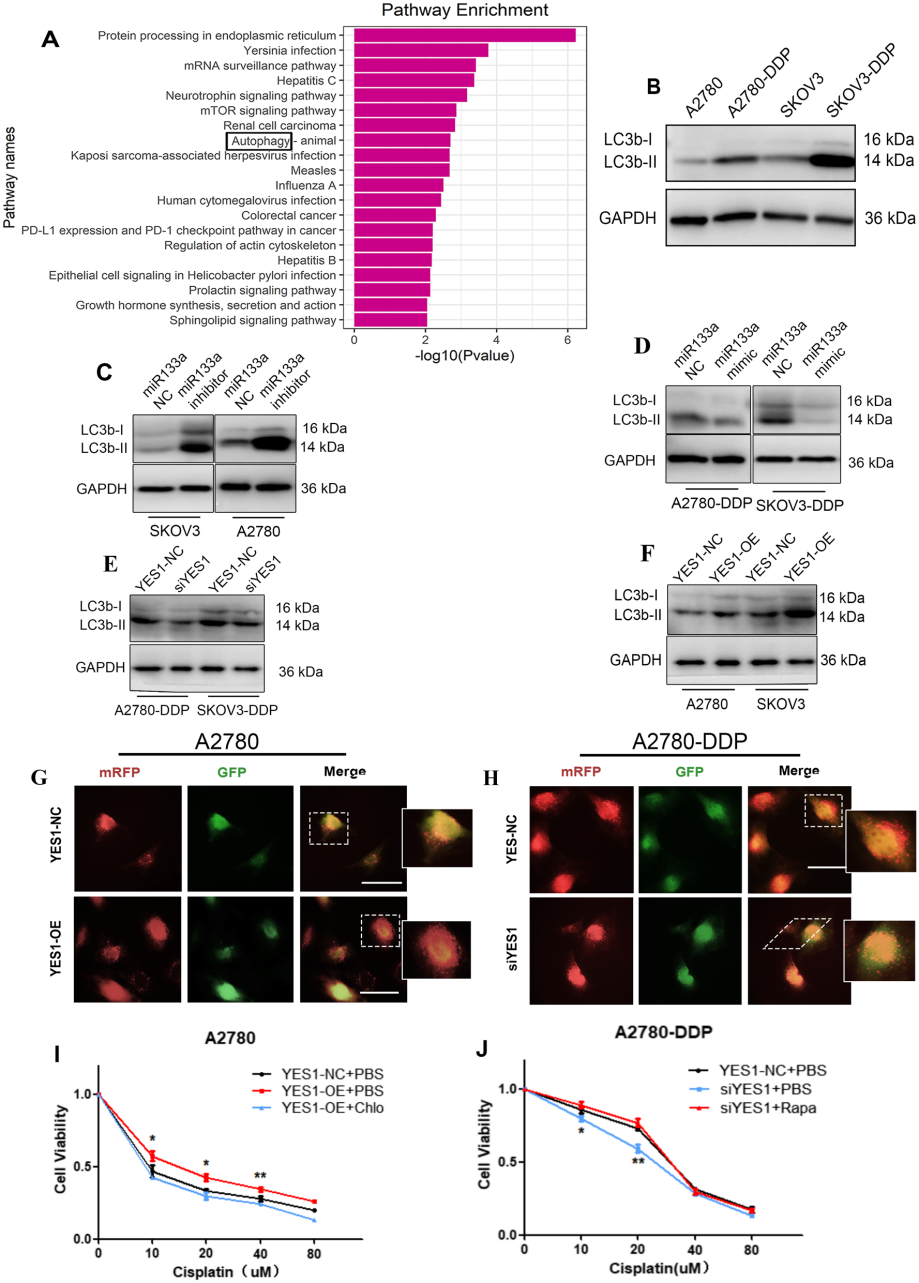
YES1 regulates autophagy in cisplatin-resistant ovarian cancer cells. **A** KEGG pathway enrichment analysis of the top 500 genes with high YES1 expression in the ovarian cancer cohort from TCGA database. **B** LC3B expression was increased in both A2780-DDP and SKOV3-DDP cells compared with A2780 and SKOV3 cells. **C** Inhibiting miR-133a significantly enhanced LC3B expression in SKOV3 and A2780 cells. **D** Transfection with the miR-133a mimic reduced LC3B expression in A2780-DDP and SKOV3-DDP cells. **E** YES1 knockdown reduces LC3B expression in SKOV3-DDP and A2780-DDP cells. **F** YES1-OE increases LC3B expression in SKOV3 and A2780 cells. **G** Autophagic flux was enhanced after transfection with mRFP-GFP-LC3 adenovirus in YES1-OE A2780 cells. **H** Autophagic flux was decreased after transfection with mRFP-GFP-LC3 adenovirus in siYES1 A2780-DDP cells. **I** Autophagic antagonist-chloroquine reverses cisplatin resistance caused by YES1-OE in A2780 cells. **J** The autophagy agonist rapamycin weakens cisplatin sensitivity caused by siYES1 in A2780-DDP cells. For comparisons, Student’s t test was performed; *p < 0.05, **p < 0.01

To further understand how YES1 functions in cisplatin resistance in ovarian cancer, the top 500 genes whose mRNA expression correlated with YES1 were identified from the RNA-seq data of the Cancer Genome Atlas (TCGA) ovarian cancer cohort (Pearson correlation p value < 0.05). Kyoto Encyclopedia of Genes and Genomes (KEGG) pathway analysis was used to identify pathways for these correlated genes. Among all of the significantly enriched pathways, the autophagy signalling pathway was the focus of the present study due to its close association with cancer (Fig. 5A). The results indicated that the autophagy signalling pathway was markedly enhanced in ovarian cancer patients with high YES1 expression. To verify these results, we first confirmed that autophagy was enhanced in both A2780-DDP and SKOV3-DDP cells compared with A2780 and SKOV3 cells (Fig. 5B). In addition, inhibiting miR-133a in SKOV3 and A2780 cells significantly enhanced autophagy (Fig. 5C), and transfecting the miR-133a mimic into A2780-DDP and SKOV3-DDP cells reduced autophagy (Fig. 5D). We further validated the relationship between Yes1 expression and autophagy. As shown in Fig. 5E, Yes1 knockdown remarkably reduced LC3B expression in A2780-DDP and SKOV3-DDP cells, and Yes1-OE remarkably increased LC3B expression in A2780 and SKOV3 cells (Fig. 5F). Autophagic flux determination also verified that Yes1 overexpression could enhance autophagic flux in A2780 cells (Fig. 5G), and Yes1 knockdown could decrease autophagic flux in A2780-DDP cells (Fig. 5H). Furthermore, the Yes1 overexpression-induced cisplatin resistance effect could be reversed by an autophagic antagonist-chloroquine (Fig. 5I). Conversely, Yes1 knockdown increased cisplatin sensitivity, and this effect was reversed by the autophagic agonist rapamycin (Fig. 5J). Taken together, these findings suggest that YES1 might affect cisplatin resistance by regulating cell autophagy.

### YES1 overexpression induces cisplatin resistance by regulating autophagy in vivo

**Fig. 6:**
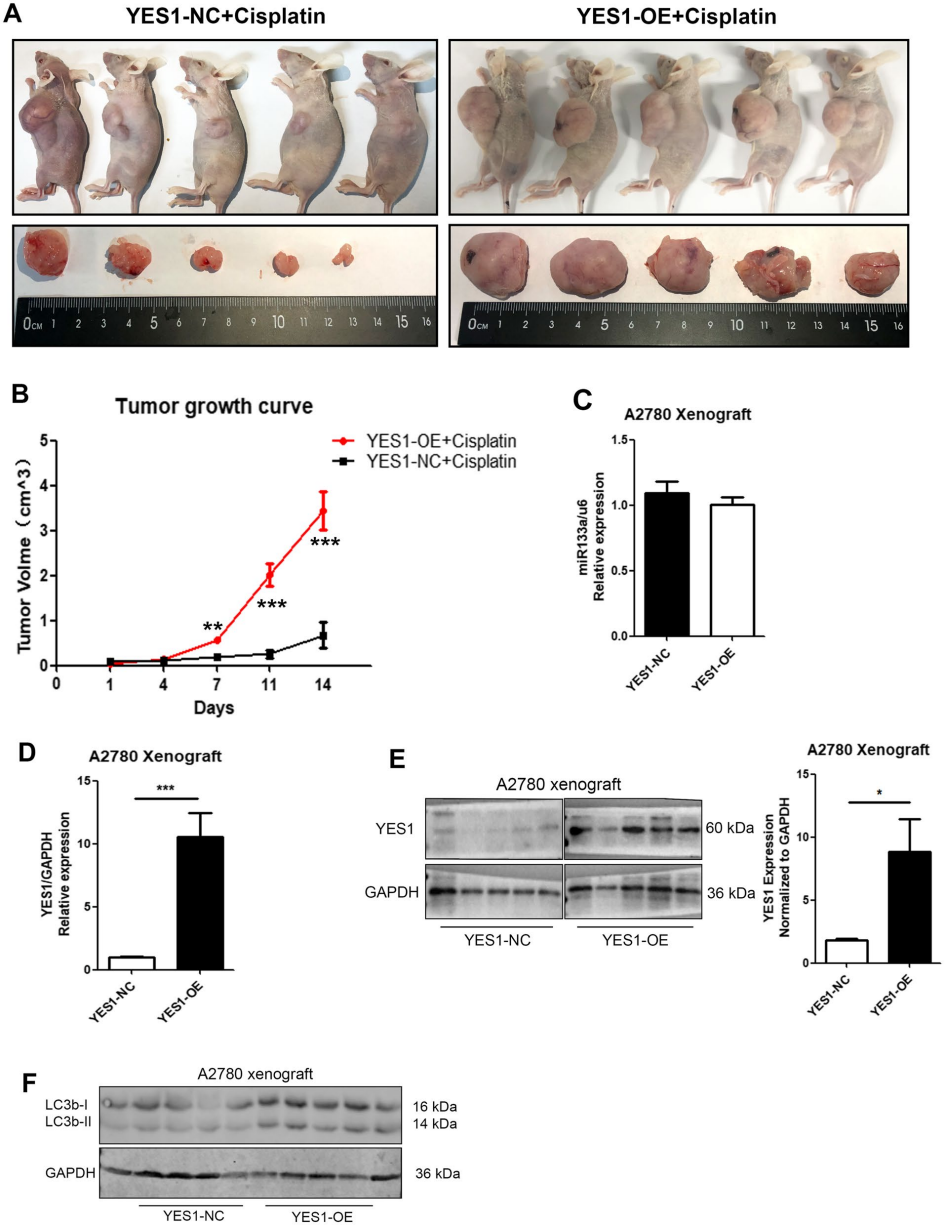
YES1 overexpression induces cisplatin resistance by regulating autophagy in vivo. **A** Nude mice and ovarian tumour tissues from the YES1-NC and YES1-OE groups at Day 14 after cisplatin injection intraperitoneally. **B** Growth curves of tumours were determined based on tumour size, which was measured every 3 Days (n = 5), and it showed significant differences in tumour volume at Days 7, 11 and 14. **C** The miR-133a expression showed no difference between the YES1-NC and the YES1-OE groups. **D** and **E** The YES1 expression increased at both the mRNA (**D**) and protein levels (**E**) in the YES1-OE group compared with the YES1-NC group. **F** LC3B expression level of xenograft tumours in the YES1-NC group and YES1-OE group after cisplatin treatment. For comparisons, Student’s t test was performed; *p < 0.05, **p < 0.01, ***p < 0.001

To further investigate the function of YES1 in cisplatin resistance of ovarian cancer in vivo, a xenograft tumour model was established by implanting A2780 ovarian cancer cells transfected with YES1-OE or YES1-NC into nude mice. As shown in Fig. 6A, Yes1 overexpression significantly enhanced ovarian tumour growth and induced cisplatin resistance, with a slower tumour growth curve and slower tumour growth volume after cisplatin injection intraperitoneally in the YES1-NC group than in the YES1-OE group (Fig. 6B). Furthermore, we examined miR-133a, YES1 and LC3B expression in mouse ovarian tumour tissues, and the results showed no difference in miR-133a expression between the YES1-NC and YES1-OE groups by qPCR (Fig. 6C) but increased expression of YES1 and LC3B in the YES1-OE group after cisplatin injection (Fig. 6D and E). In summary, we demonstrated that YES1 overexpression enhanced cisplatin resistance by activating cell autophagy in vivo.

## Discussion

Currently, chemotherapy resistance in both primary and recurrent ovarian cancer contributes to poor prognosis and high mortality. Recently, research on tumour-associated miRNAs has received increasing attention. Emerging evidence has suggested that miR-133a plays a critical role in different kinds of cancers [5]. Some studies have proven that miR-133a promotes tumour development in multiple cancers by regulating autophagy. For example, miR-133a could target GABA type A receptor associated protein like 1 (GABARAPL1) to inhibit autophagy-mediated glutaminolysis, repressing gastric cancer growth and metastasis [8]. MiR-133a could also regulate cell autophagy by binding to the 3′UTR of fork head Box P3 (FOXP3**)** in gastric cancer [18]. Moreover, miR-133a also plays a key role in tumour drug resistance. MiR-133a negatively regulated ATPase copper transporting beta (ATP7B) expression, reducing cisplatin resistance in Hep-2v cells [11]. Overexpression of miR-133a could decrease doxorubicin resistance in the MCF-7/Dox breast cancer cell line by decreasing the expression of mitochondrial uncoupling protein 2 (UCP-2) [10]. Recently, Li et al. reported that miR-133a played a role in reducing cisplatin resistance in non-small-cell lung cancer through endogenous competition with lncRNA SNHG14 [12]. However, the function of miR-133a in ovarian cancer chemotherapy resistance has not been researched. Here, we first demonstrated that miR-133a reduced cisplatin resistance in ovarian cancer cells. We revealed that miR-133a expression was significantly decreased in cisplatin-resistant ovarian cancer cell lines. MiR-133a inhibitor increased cisplatin resistance, and miR-133a mimic treatment increased cisplatin sensitivity in vitro. Furthermore, we identified miR-133a as a potential YES1 posttranscriptional regulator that directly binds the 3′UTR of YES1 and downregulates its expression in ovarian cancer. Multiple studies have demonstrated that YES1 functions as an oncogene and is dysregulated in various carcinomas [13]. Previous research has reported that miR-133a inhibits cell proliferation in non-small-cell lung cancer by targeting YES1 [19]. In this study, we further investigated the roles of YES1, a protein highly expressed in cisplatin-resistant tissue and cell lines, in cisplatin-resistant ovarian cancer. Our results also demonstrate that YES1 knockdown suppresses cisplatin resistance in ovarian cancer in vitro and that YES1 overexpression induces cisplatin resistance both in vitro and in vivo.

Previous studies have also confirmed that inhibition of autophagy could increase the sensitivity to cisplatin of ovarian cancer [20, 21]. Overexpression of miR-133a inhibits autophagy by downregulating ras-related C3 botulinum toxin substrate 1 (RAC1) in Parkinson’s disease in vitro [22]. Moreover, inhibition of lncRNA XIST could improve myocardial I/R injury by targeting miR-133a through inhibition of autophagy [23]. In this study, we analysed the Cancer Genome Atlas (TCGA) ovarian cancer cohort to identify the YES1 signature as the top 500 YES1-correlated genes (Pearson correlation P value <0.05). The signature genes were extracted for Kyoto Encyclopedia of Genes and Genomes (KEGG) pathway enrichment and found to be enriched in the autophagy signalling pathway (Fig. 2A). Furthermore, we first verified that knockdown of Yes1 inhibited the expression of LC3B and decreased autophagic flux, and overexpression of Yes1 activated LC3B expression and increased autophagic flux in vitro. Moreover, rapamycin, an autophagic agonist, reversed siYes1-induced cisplatin resistance, and chloroquine, an autophagy antagonist, reversed Yes1-OE-induced cisplatin resistance (Fig. 5I, J), which further demonstrated that YES1 affects cisplatin resistance in ovarian cancer by regulating cell autophagy. Overexpression of Yes1 also increased LC3B expression and led to cisplatin resistance in a xenograft tumour model (Fig. 6).

Additionally, this study has some limitations. For example, qRT-PCR, CCK-8 and other experiments in this study proved that miR-133a plays an important role in drug-resistant ovarian cancer, but whether miR-133a is the only miRNA responsible for the chemo-resistance in ovarian cancer was still not fully verified. Moreover, we studied whether YES1 regulates cisplatin resistance in ovarian cancer. Previous studies proved that some of the most important pathways, such as the AMPK, Hippo and mTOR pathways, may participate in the regulatory roles of autophagy [24–26], but our study did not definitively verify whether YES1 plays a role by influencing these important pathways, which is also worth studying. Additionally, in our study, we only demonstrated that YES1 was upregulated in recurrent tissues resistant to platinum chemotherapy by bioinformatics analysis and did not prove the results by our own clinical specimens.

Taken together, our research suggested that miR-133a directly targets YES1 by binding its 3′UTR area and that the miR-133a/YES1 axis might regulate cisplatin sensitivity via autophagy in ovarian cancer. Our findings provide insight into the miR-133a/YES1/autophagy axis as a novel diagnostic biomarker and potential gene therapeutic target for ovarian cancer chemotherapy.

## Supplementary Information

**Additional file 1.** **Figure S1**. Among the 5 potential genes, the YES1 expression level was dramatically increased in the relapse group compared with the primary sensitive group. **Figure S2**. SiRNA2 sequence showed highest interference efficiency.

## Data Availability

The datasets obtained and analysed during the current study were made available from the corresponding authors through request.
