# Supplementary material for: miR-133a targets YES1 to reduce cisplatin resistance in ovarian cancer by regulating cell autophagy
Source: Cancer Cell Int. 2022 Jan 10;22:15. doi: 10.1186/s12935-021-02412-x (PMC8751326; doi:10.1186/s12935-021-02412-x)
Supplement: Supplementary file 1 — Additional file 1. Figure S1. Among the 5 potential genes, the YES1 expression level was dramatically increased in the relapse group compared with the primary sensitive group. Figure S2. SiRNA2 sequence showed highest interference efficiency. [file 12935_2021_2412_MOESM1_ESM.docx]

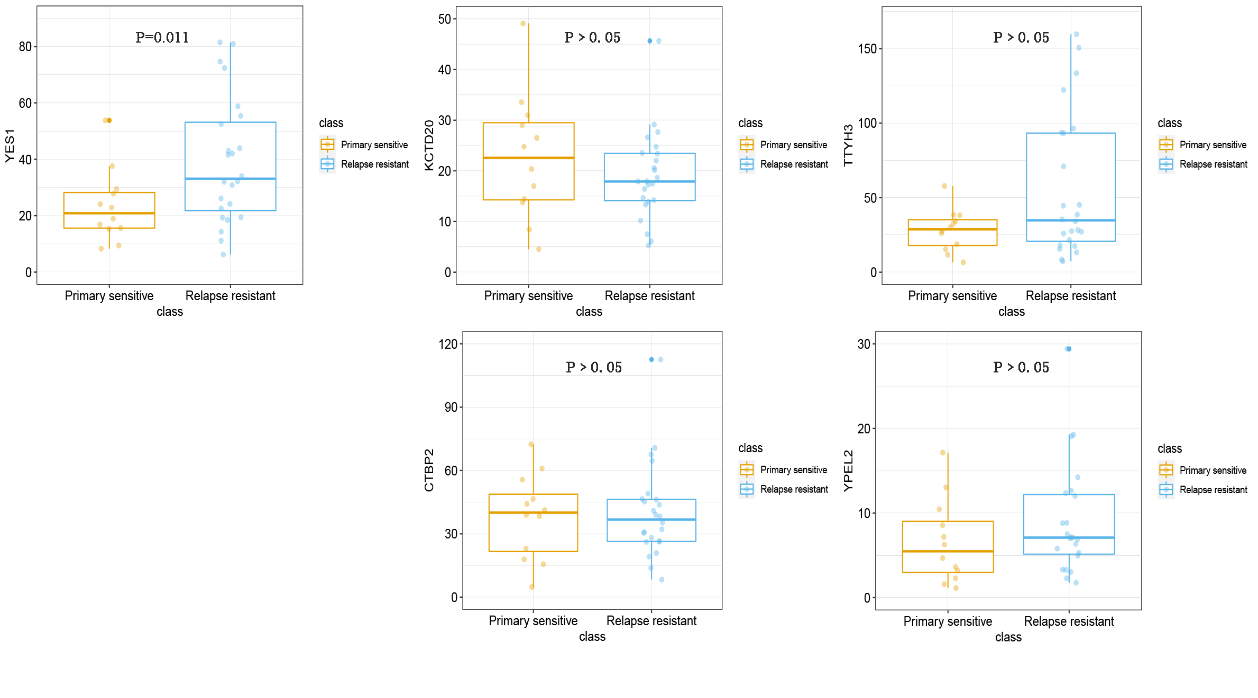
**Figure S1.** The expression levels of YES1, KCTD20,TTYH3,CTBP2 and YPEL2 between primary ovarian cancer and recurrent ovarian cancer groups from the RNA sequencing data of ICGC dataset analysis.

Among the 5 potential genes, only the YES1 expression level was increase in the relapse group compared with the primary sensitive group.

**Figure S2.** The interference efficiency of five YES1 siRNAs with different targeting sequences.

The siRNA2 sequence showed highest interference efficiency.

| si1 | GTCATTATTTCCTCTTATC |
| --- | --- |
| si2 | ACCACGAAAGTAGCAATCA |
| si3 | GCAAGGTTAATTGAAGACA |
| si4 | AGACTCAAGGTCTAGCAAA |
| si5 | GTGACAATGTGAAACACTA |

NC ATGCGGCCTATGCGTTGAG

**Table 1.** Interfering RNA (siRNA) sequences for YES1.
